# Sequencing antibody-drug conjugates in metastatic cancer (Review)

**DOI:** 10.3892/mi.2026.308

**Published:** 2026-03-09

**Authors:** Berun A. Abdalla

**Affiliations:** 1 Kscien Organization for Scientific Research (Middle East Office), Sulaymaniyah 46001, Iraq; 2 Department of Scientific Affairs, Smart Health Tower, Sulaymaniyah 46001, Iraq

**Keywords:** antibody-drug conjugates, metastatic cancer, treatment sequencing, cross-resistance, targeted therapy, payload swap

## Abstract

The therapeutic landscape of metastatic solid tumors has been fundamentally reshaped by the rapid development and regulatory approval of multiple antibody-drug conjugates (ADCs) across breast, urothelial and lung cancers. As these targeted agents move into earlier lines of therapy, patients are experiencing prolonged survival and are increasingly becoming candidates for sequential ADC treatment. This evolution introduces a critical and complex clinical dilemma: The question of whether a second ‘Trojan Horse’ remains effective following the failure of a prior ADC, or whether shared resistance mechanisms undermine sequential efficacy. While the expanding ADC armamentarium provides new therapeutic opportunities, emerging real-world data suggest that sequential use is frequently complicated by cross-resistance, particularly when successive agents employ similar cytotoxic payloads such as topoisomerase I inhibitors. The present narrative review critically discusses the biological basis of resistance in the sequential ADC setting. The present review distinguishes between target-mediated resistance, in which antigen downregulation limits drug delivery, and payload-mediated resistance, in which intracellular mechanisms neutralize the cytotoxic component. By synthesizing clinical evidence from metastatic breast and urothelial carcinoma cohorts, the present review highlights the diminished efficacy often observed when changing targets without altering payload class. Therefore, a pragmatic sequencing framework emphasizing ‘payload swapping’ is proposed, prioritizing a change in cytotoxic mechanisms over antigen switching to optimize patient outcomes and minimize futile toxicity in this rapidly evolving therapeutic era.

## 1. Introduction

The field of oncology is currently experiencing an unprecedented expansion in the development and clinical adoption of antibody-drug conjugates (ADCs). Following the foundational success of ado-trastuzumab emtansine (T-DM1), multiple next-generation ADCs targeting human epidermal growth factor receptor 2 (HER2), trophoblast cell-surface antigen 2 (TROP2), Nectin-4 and MET have received regulatory approval in recent years (1). These agents have rapidly become integral components of standard-of-care treatment algorithms for metastatic breast cancer, urothelial carcinoma and non-small cell lung cancer (NSCLC).

This rapid expansion has introduced a novel and clinically relevant dilemma. Historically, disease progression in the metastatic setting prompted a switch between chemotherapy classes (e.g., taxane to platinum). By contrast, contemporary practice increasingly involves transitioning from one ADC to another. This paradigm shift raises a fundamental question: Whether the ‘Trojan Horse’ delivery strategy remains effective following prior ADC exposure, or whether shared resistance mechanisms render subsequent agents ineffective (2).

The clinical implications of this uncertainty are substantial. Inappropriate sequencing may expose patients to costly and potentially toxic therapies with limited likelihood of benefit. For example, in the event that resistance develops primarily against the cytotoxic payload of an initial ADC, a subsequent agent carrying a similar warhead may exhibit immediate cross-resistance, regardless of its antigenic target (3). Accordingly, a more in-depth understanding of resistance mechanisms is essential to guide rational sequencing decisions.

In addition to breast and urothelial malignancies, the present review also considers emerging sequencing challenges in NSCLC and gastric cancer, where multiple ADCs are entering clinical practice. As novel agents, such as trastuzumab deruxtecan, telisotuzumab vedotin and datopotamab deruxtecan become integrated into treatment algorithms, understanding payload-driven resistance and rational ADC transition strategies across tumor types has become increasingly important. Accordingly, the present review adopts a tumor-agnostic framework focused on shared biological mechanisms of resistance while incorporating disease-specific clinical evidence where available (4,5).

The present narrative review discusses the biological drivers of resistance to sequential ADC therapy, with a focus on distinguishing target-mediated resistance (failure of antigen recognition and internalization) from payload-mediated resistance (failure of intracellular cytotoxic activity). This framework is intended to provide clinicians with a practical approach to navigating an increasingly complex therapeutic landscape.

## 2. Mechanisms of resistance

### Antigen downregulation

The efficacy of any ADC is contingent upon the sufficient expression of its target antigen on the tumor cell surface. A well-recognized mechanism of resistance is antigen downregulation or loss, effectively preventing ADC binding and internalization. In HER2-positive breast cancer, treatment with trastuzumab deruxtecan (T-DXd) has been shown to select for HER2-low or HER2-null tumor clones (6). When antigen expression falls below a critical threshold, subsequent ADCs targeting the same antigen, such as switching from T-DXd to trastuzumab emtansine (T-DM1) are unlikely to be effective because the delivery vehicle can no longer reliably engage the tumor cell (6).

### Bystander effect and primary vs. acquired resistance in ADC sequencing

An additional consideration in ADC sequencing is the role of the bystander effect, particularly for ADCs carrying membrane-permeable, high-potency payloads such as T-DXd. The bystander effect may partially mitigate heterogeneous or reduced antigen expression by allowing payload diffusion into adjacent antigen-low or antigen-negative tumor cells. This property likely contributes to the clinical activity of T-DXd in HER2-low tumors. However, available data suggest that the bystander effect does not fully overcome resistance once antigen expression falls below a critical threshold or when resistance is driven predominantly by payload-specific mechanisms such as drug efflux, altered intracellular trafficking, or DNA (deoxyribonucleic acid) damage response adaptation. In these contexts, the sequential use of ADCs sharing the same payload class may still result in diminished efficacy despite preserved bystander activity (7,8).

Notably, resistance to ADCs can be broadly categorized as primary (intrinsic) or acquired. Primary resistance reflects pre-existing tumor features, including absent or heterogeneous antigen expression, intrinsic insensitivity to the payload, or baseline efflux activity, and may limit the effectiveness of any ADC targeting the same pathway. By contrast, acquired resistance arises following ADC exposure and is frequently driven by adaptive changes such as antigen downregulation, payload-specific resistance mechanisms, or alterations in intracellular processing. This distinction has practical implications for sequencing: patients with acquired, antigen-specific resistance may remain candidates for a rational ‘target swap’ or ‘payload swap’, whereas those with primary payload-class resistance are less likely to benefit from sequential ADCs carrying similar cytotoxic warheads (8). While the loss of HER2 represents the most well-characterized example of target-mediated resistance, antigen downregulation in non-HER2 targets, such as TROP2 and Nectin-4 remains less clearly defined, and ongoing translational and clinical studies are expected to further clarify its role in ADC resistance and sequencing decisions.

### Payload cross-resistance

Even when antigen binding and internalization remain intact, resistance to the cytotoxic payload can lead to therapeutic failure. A major concern in contemporary practice is the increasing reliance on camptothecin-derived topoisomerase I inhibitors as ADC payloads. Agents such as trastuzumab deruxtecan, sacituzumab govitecan and datopotamab deruxtecan all share this mechanism of action (9).

In the event that a tumor acquires resistance to one topoisomerase I inhibitor through target enzyme mutations, alterations in DNA damage response pathways, or enhanced drug efflux it is biologically plausible that cross-resistance will extend to other ADCs carrying similar payloads. In addition, cancer cells may upregulate ATP-binding cassette transporters, including multidrug resistance 1 (P-glycoprotein), which actively export cytotoxic agents from the cytoplasm before they can induce lethal DNA damage or microtubule disruption (10). Such mechanisms can confer broad resistance across multiple ADCs, irrespective of target antigen.

### Linker and intracellular processing defects

Resistance may also arise from the impaired intracellular processing of the ADC. Following internalization, linker cleavage within the lysosome is required to release the active payload. Lysosomal dysfunction, such as increased lysosomal pH or reduced expression of proteolytic enzymes (e.g., cathepsin B) can prevent effective payload release (11). In this context, tumor cells internalize the ADC, but fail to liberate the cytotoxic component, resulting in resistance that may extend across ADCs utilizing similar linker technologies. Although linker instability and intracellular processing defects represent biologically plausible mechanisms of resistance, current evidence supporting their role is derived largely from preclinical and translational studies, and their relative clinical impact appears less well established compared with antigen downregulation or payload-mediated resistance.

## 3. Clinical evidence

### Sequencing in breast cancer

Breast cancer provides the most robust clinical data regarding ADC sequencing and highlights the importance of payload diversity. The sequence of T-DM1 followed by T-DXd (topoisomerase I inhibitor payload) has demonstrated substantial clinical benefit. The phase III DESTINY-Breast03 trial established the superiority of T-DXd over T-DM1 in patients with previously treated HER2-positive metastatic breast cancer, demonstrating significantly prolonged progression-free survival and higher response rates (12). The superiority of T-DXd over T-DM1 has been demonstrated, and real-world data confirm its continued efficacy following T-DM1 failure (8). This success is likely attributable to both a ‘payload swap’ and the higher drug-to-antibody ratio of T-DXd.

By contrast, sequencing T-DXd (HER2-targeted) followed by sacituzumab govitecan (TROP2-targeted) represents a ‘target swap’ without a ‘payload swap’, as both ADCs deliver topoisomerase I inhibitors. Emerging retrospective and real-world cohort data, including the multi-institutional analysis by Mai *et al*, suggest diminished efficacy when a topoisomerase I-based ADC is administered after progression on a prior agent with the same payload class (13). These findings are hypothesis-generating and require prospective validation. A recent multi-institutional retrospective analysis reported shorter progression-free survival among patients receiving a topoisomerase I-based ADC after progression on a prior agent with the same payload class, compared with payload-naïve patients (13). These findings strongly support the clinical relevance of payload-mediated cross-resistance. It is important to note that these observational sequencing data are retrospective in nature and inherently subject to selection bias and confounding, and therefore should be interpreted as complementary to, rather than a replacement for, evidence derived from prospective randomized clinical trials.

### Sequencing in urothelial cancer

In metastatic urothelial carcinoma, the standard sequencing paradigm typically involves enfortumab vedotin (EV), which carries a monomethyl auristatin E (MMAE) microtubule-disrupting payload, followed by sacituzumab govitecan in subsequent lines of systemic therapy (14,15). This sequence generally preserves clinical activity, likely as the payloads disrupt distinct cellular processes. The relative absence of cross-resistance in this setting further supports the hypothesis that switching cytotoxic mechanisms rather than targets alone is critical for maintaining efficacy in sequential ADC therapy (14,15). However, it should be emphasized that sequencing data in metastatic urothelial carcinoma remain evolving, with additional ADCs and combination strategies currently under investigation, and therefore current conclusions should be interpreted within the context of a rapidly developing therapeutic landscape.

### Sequencing considerations in NSCLC and gastric cancer

Emerging data from NSCLC and gastric cancer further support the importance of payload class in determining clinical benefit. In HER2-mutant and HER2-overexpressing NSCLC, T-DXd, a topoisomerase I inhibitor-based ADC, has demonstrated substantial activity and is currently an established therapeutic option. However, as additional ADCs enter clinical practice, such as telisotuzumab vedotin (MET-directed, MMAE payload) and datopotamab deruxtecan (TROP2-directed, topoisomerase I payload) sequencing considerations analogous to those observed in breast cancer are emerging. Early-phase clinical trials and emerging clinical data in NSCLC, including the LUMINOSITY study (telisotuzumab vedotin) and the phase III TROPION-Lung01 trial (datopotamab deruxtecan), suggest that preserved efficacy may be more likely when transitioning between ADCs with distinct cytotoxic payload classes, whereas sequential exposure to ADCs sharing topoisomerase I inhibitor payloads may be associated with reduced benefit due to payload-driven cross-resistance (4,16). Similar principles may apply to gastric cancer, where trastuzumab deruxtecan demonstrated improved overall survival and response rates compared with chemotherapy in previously treated HER2-positive metastatic gastric cancer in the phase II DESTINY-Gastric01 trial, underscoring the broader relevance of payload diversification rather than target substitution alone in rational ADC sequencing strategies (5).

## 4. Strategic considerations for the clinician

### The ‘payload swap’ strategy

**Figure 1:**
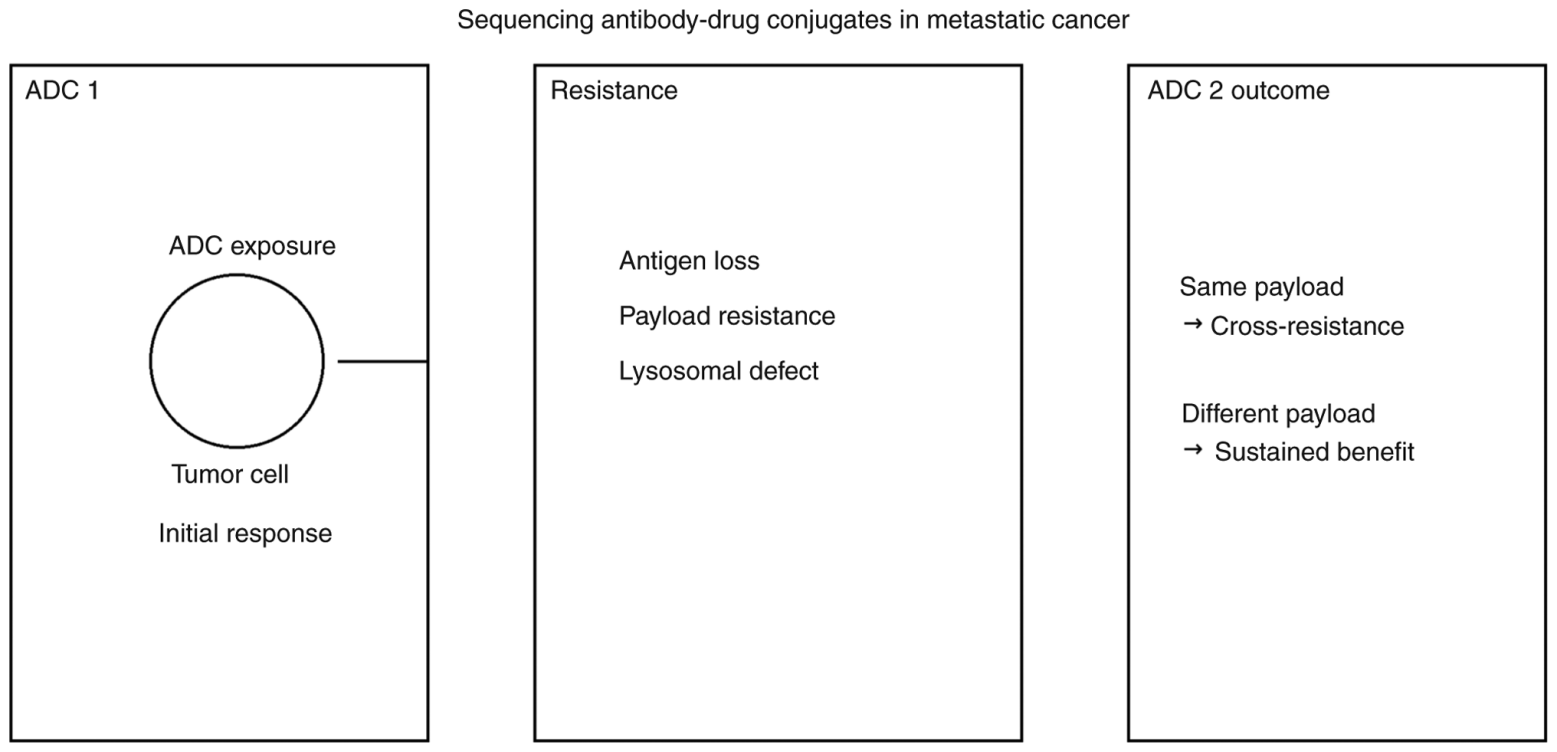
Conceptual framework illustrating the sequencing of ADCs in metastatic cancer. ADC, antibody-drug conjugate.

Emerging clinical and translational data, as summarized in a recent review, suggest that prioritizing a change in payload class may represent a rational sequencing approach (6). When progression occurs on an ADC, subsequent therapy should ideally employ a cytotoxic mechanism distinct from the first. For example, transitioning from a microtubule inhibitor-based ADC (e.g., T-DM1 or EV) to a topoisomerase I inhibitor-based ADC (e.g., T-DXd or sacituzumab govitecan) appears to reduce the risk of cross-resistance (Table I) (13). These mechanisms collectively illustrate how resistance acquired during initial ADC exposure can influence the efficacy of subsequent ADCs, highlighting the importance of payload selection in sequencing strategies (Fig. 1). It should be emphasized that this proposed ‘payload swap’ strategy is supported primarily by indirect and retrospective clinical evidence and is intended to guide mechanistic clinical reasoning rather than serve as a definitive treatment guideline, pending validation in prospective sequencing trials.

Beyond efficacy considerations, overlapping payload-associated toxicities represent an additional practical constraint in ADC sequencing. For example, sequential use of DXd-based ADCs (e.g., trastuzumab deruxtecan or datopotamab deruxtecan) may increase clinical vigilance for interstitial lung disease (ILD), whereas back-to-back exposure to MMAE-based ADCs (e.g., trastuzumab emtansine or enfortumab vedotin) may compound the risk of cumulative peripheral neuropathy. In this context, a ‘payload swap’ may not only mitigate biological cross-resistance but also alter the toxicity profile, potentially improving tolerability in selected patients. Therefore, sequencing decisions should integrate both mechanistic resistance principles and anticipated safety overlap (12,14,16).

### The ‘target swap’ strategy

Switching antigen targets while retaining a similar payload class (e.g., T-DXd to sacituzumab govitecan) carries a higher risk of diminished efficacy. Although a new antigen may permit cellular entry, intracellular resistance to the cytotoxic warhead may limit the depth and duration of response (9). Clinicians should approach such sequences cautiously and counsel patients regarding the potential for reduced benefit. In general, a target swap strategy may be most appropriate in patients with confirmed preservation of the new target antigen on repeat biopsy, limited alternative treatment options, adequate organ function, and no clear evidence of payload-class resistance from prior therapy.

### The role of re-biopsy

Given the prevalence of antigen downregulation, obtaining a repeat biopsy prior to initiating a second ADC is increasingly important. Confirming adequate expression of the intended target antigen (e.g., HER2 or TROP2) can help avoid target-mediated resistance and guide rational treatment selection (6). However, re-biopsy may be constrained by procedural risks, limited access in certain practice settings, financial cost, and feasibility in heavily pre-treated patients, and therefore should be pursued selectively when the anticipated clinical benefit justifies these considerations.

## 5. Limitations

The present review has several critical limitations that should be acknowledged. First, as a narrative review, the manuscript does not follow a systematic review or meta-analytic methodology. Consequently, the included studies were selected based on clinical relevance and emerging impact rather than predefined inclusion criteria, and the conclusions should be interpreted as hypothesis-generating rather than definitive.

Second, much of the clinical evidence informing ADC sequencing, particularly regarding payload cross-resistance, is derived from retrospective analyses, real-world cohorts and subgroup observations, which are inherently subject to selection bias and confounding. While these data provide valuable insights into real-world practice, they cannot fully substitute for prospective, randomized sequencing trials, which remain limited or unavailable in most tumor types.

Third, although the review emphasizes payload-mediated resistance as a dominant driver of sequential ADC failure, direct comparative data between different sequencing strategies are scarce, and resistance mechanisms are often inferred from translational or preclinical studies. The relative contribution of antigen loss, payload resistance, linker instability, and intracellular trafficking defects likely varies across tumor types and individual patients, limiting the generalizability of any single sequencing paradigm.

Finally, the rapidly evolving ADC landscape represents an inherent limitation. New ADCs, novel payload classes, and emerging clinical trial data (including conference abstracts) continue to reshape sequencing considerations, and recommendations discussed here may require refinement as prospective evidence matures.

## 6. Future directions and conclusion

Next-generation ADC strategies are currently under active investigation and aim to overcome limitations observed with existing sequencing paradigms. Bispecific ADCs targeting two antigens simultaneously (e.g., HER2 and EGFR) are being developed to address tumor heterogeneity and potential antigen loss. In addition, novel payload classes, including immunostimulatory agents such as stimulator of interferon genes agonists, as well as dual-payload constructs, are being explored to circumvent established resistance pathways.

Notably, these approaches remain investigational and are largely supported by preclinical data or early-phase clinical studies. While conceptually promising, their safety, durability of response, and optimal integration into existing sequencing strategies have yet to be defined. Accordingly, the clinical utility of these next-generation ADC platforms remains to be established through prospective clinical trials.

In conclusion, sequential ADC therapy has become a cornerstone of modern metastatic cancer management, but carries a substantial risk of futility if cross-resistance is not carefully considered. Accumulating evidence indicates that resistance is more commonly driven by the cytotoxic payload than by the target antigen. Consequently, successful sequencing often depends on prioritizing a ‘payload swap’ rather than merely changing targets. Incorporating this mechanistic approach into clinical decision-making is essential to maximize therapeutic benefit while minimizing unnecessary toxicity in the evolving ADC era.

## Figures and Tables

**Table I:** Antibody-drug conjugates by target and payload class.

ADC	Target	Payload class	Toxicities
Trastuzumab emtansine (T-DM1)	HER2	Microtubule inhibitor	Thrombocytopenia, hepatotoxicity, peripheral neuropathy
Trastuzumab deruxtecan	HER2	Topoisomerase I inhibitor	ILD/pneumonitis, nausea
Sacituzumab govitecan	TROP2	Topoisomerase I inhibitor	Neutropenia, diarrhea
Enfortumab vedotin	Nectin-4	Microtubule inhibitor	Peripheral neuropathy, skin toxicity
Datopotamab deruxtecan	TROP2	Topoisomerase I inhibitor	ILD/pneumonitis, stomatitis

ADC, antibody-drug conjugate; ILD, interstitial lung disease.

## Data Availability

Not applicable.
